# HO-1 Interactors Involved in the Colonization of the Bone Niche: Role of ANXA2 in Prostate Cancer Progression

**DOI:** 10.3390/biom10030467

**Published:** 2020-03-18

**Authors:** Nicolás Anselmino, Juan Bizzotto, Pablo Sanchis, Sofia Lage-Vickers, Emiliano Ortiz, Pia Valacco, Alejandra Paez, Estefania Labanca, Roberto Meiss, Nora Navone, Javier Cotignola, Elba Vazquez, Geraldine Gueron

**Affiliations:** 1 Universidad de Buenos Aires, Facultad de Ciencias Exactas y Naturales, Departamento de Química Biológica, Laboratorio de Inflamación y Cáncer, Buenos Aires C1428EGA, Argentina; nicoanselmino@gmail.com (N.A.); juanantoniobizzotto@gmail.com (J.B.); pabloasanchis@gmail.com (P.S.); sofilage@gmail.com (S.L.-V.); emilianogortiz@gmail.com (E.O.); pvalacco@qb.fcen.uba.ar (P.V.); jcotignola@qb.fcen.uba.ar (J.C.); 2 CONICET - Universidad de Buenos Aires, Instituto de Química Biológica de la Facultad de Ciencias Exactas y Naturales (IQUIBICEN), Buenos Aires C1428EGA, Argentina; 3 Unidad de Transferencia Genética, Instituto de Oncología “Angel H Roffo”, Universidad de Buenos Aires, Buenos Aires C1417DTB, Argentina; alejandravpaez@gmail.com; 4 Department of Genitourinary Medical Oncology and the David H. Koch Center for Applied Research of Genitourinary Cancers, The University of Texas MD Anderson Cancer Center, Houston, TX 77030, USA; estefania.labanca@gmail.com (E.L.); nnavone@mdanderson.org (N.N.); 5 Academia Nacional de Medicina, Buenos Aires C1425ASU, Argentina; rpmeiss@gmail.com

**Keywords:** heme-oxygenase-1 (HO-1), prostate cancer (PCa), annexin A2 (ANXA2), bone, metastasis, urokinase-plasminogen activator (uPA)

## Abstract

Background: Prostate cancer (PCa) dissemination shows a tendency to develop in the bone, where heme oxygenase 1 (HO-1) plays a critical role in bone remodeling. Previously by LC/ESI-MSMS, we screened for HO-1 interacting proteins and identified annexin 2 (ANXA2). The aim of this study was to analyze the relevance of ANXA2/HO-1 in PCa and bone metastasis. Methods: We assessed *ANXA2* levels using a co-culture transwell system of PC3 cells (pre-treated or not with hemin, an HO-1 specific inducer) and the pre-osteoclastic Raw264.7 cell line. Results: Under co-culture conditions, *ANXA2* mRNA levels were significantly modulated in both cell lines. Immunofluorescence analysis unveiled a clear ANXA2 reduction in cell membrane immunostaining for Raw264.7 under the same conditions. This effect was supported by the detection of a decrease in Ca^2+^ concentration in the conditioned medium. HO-1 induction in tumor cells prevented both, the ANXA2 intracellular relocation and the decrease in Ca^2+^ concentration. Further, secretome analysis revealed urokinase (uPA) as a key player in the communication between osteoclast progenitors and PC3 cells. To assess the clinical significance of ANXA2/HO-1, we performed a bioinformatics analysis and identified that low expression of each gene strongly associated with poor prognosis in PCa regardless of the clinico-pathological parameters assessed. Further, these genes appear to behave in a dependent manner. Conclusions: ANXA2/HO-1 rises as a critical axis in PCa.

## 1. Introduction

Prostate cancer (PCa) is one of the most diagnosed cancer in men and the skeletal complications of this disease represent a difficult clinical hurdle to overcome [1,2]. PCa cells and the bone microenvironment communicate and interact with each other to develop a fertile niche for the promotion of the metastatic process [3,4,5].

Evidence has recognized inflammation as a risk factor for this neoplastic disease [6]. Heme oxygenase 1 (HO-1), encoded by the *HMOX1* gene, is a stress response protein and a critical mediator of cellular homeostasis [7]. Although the role of HO-1 in cancer is controversial [8,9], we have shown that its pharmacologic or genetic upregulation is associated with a less aggressive phenotype in PCa [10]. HO-1 impairs tumor growth and angiogenesis in vivo and downregulates the expression of target genes associated with inflammation in PCa [11,12]. In the metastatic bone site, we demonstrated that HO-1 is capable of modulating signaling pathways relevant to skeletal PCa metastasis, such as FoxO/β-catenin and promotes bone remodeling when human tumor cells are transplanted into the femur of SCID mice [13]. Moreover, we have shown the direct effect of HO-1 on bone turnover and remodeling. When assessing the physiological impact of *Hmox1* gene knockout on bone metabolism in vivo, histomorphometric analysis of *Hmox1*−/− mice bones exhibited significantly decreased bone density. A positive correlation between *Hmox1* expression and key bone markers was observed in primary mouse osteoblasts (PMOs) [14]. These observations highlight the importance of HO-1 expression in bone, not only for the physiology of bone cells but also in the modulation of the communication between PMOs and PCa cells by soluble factors [14].

We also reported that HO-1 induction alters the expression of different cytoskeletal genes and is associated with key factors that induce the remodeling of actin filaments in the cell filopodia, increasing adhesion and decreasing PCa cell invasiveness [15]. Through a multi “omics” approach we defined the HO-1 interactome in PCa cells identifying 56 molecular partners of this protein, most of them involved in cell adhesion and cell–cell communication [16]. Further, this work provided a four molecular pathway foundation (ANXA2/HMGA1/POU3F1; NFRSF13/GSN; TMOD3/RAI14/VWF; PLAT/PLAU) behind HO-1 regulation of tumor cytoskeletal cell compartments. We were particularly interested in Annexin A2 (ANXA2), as it is highly implicated in bone physiology and in PCa bone progression [3,17]. Annexins are related to several biological functions including apoptosis, membrane trafficking, signal transduction, cellular motility, cell–cell interactions, and oxidative stress regulation [18,19,20,21]. In particular, ANXA2 is a family member of calcium-dependent phospholipid membrane-binding proteins, containing a conserved repeating domain of approximately 70 amino acids. This molecule is highly abundant in lipid rafts and likely takes part in ion channel regulation and tight junction formation [3,17].

Regarding the bone compartment, ANXA2 is expressed in bone cells and acts as a chemotactic factor promoting the tumor tropism to this homing organ [3,17]. In turn, PCa cells express the ANXA2 receptor (ANXA2R), hence this might increase the migratory capacity of tumor cells to the bone [3,17]. Using bone metastases models, Shiozawa and colleagues [22], demonstrated that human PCa cells compete with hematopoietic stem cells (HSCs) for the bone marrow niche. It appears as if the expression of the ANXA2 and CCL12 (SDF-1) on marrow stromal cells and osteoblasts in the endosteal hematopoietic stem cell niche enables HSCs and PCa cells that express CXCR4 and ANXA2R to attach and colonize the marrow [22].

It is well recognized that the loss of ANXA2 expression is specific for PCa disease [23], hence the lack of ANXA2 might exert a selective pressure that favors skeletal metastasis [22]. As mentioned above, the bone is a rich source of ANXA2 [22], a molecule produced by several cells, including osteoclasts and it is involved in osteoclast formation and bone resorption [24]. Although PCa bone metastasis are mainly osteoblastic, an underlying osteoclastic component should not be overlooked [25].

To explore the contribution of HO-1 in the interaction between PCa cells and osteoclasts, our previous work documented that PCa cells exposed to conditioned medium from a co-culture system of PC3 and osteoclastic progenitor cells, displayed reduced membrane filopodia density and contact among cells, effects prevented by HO-1 induction in tumor cells [16]. Our results suggest that HO-1 prevents PCa cells from extravasation and invasion to other homing organs. If ANXA2 acts as a chemoattractant and regulates the migration and adhesion of tumor cells and HO-1 halts tumor adhesion to the bone compartment, understanding how HO-1/ANXA2 axis impacts on PCa skeletal metastasis is critical to identify the signaling cascades that govern the skeletal complication of PCa progression.

In this work, we analyzed the relevance of ANXA2/HO-1 in PCa and bone metastasis. We used a co-culture transwell system of PC3 cells and the pre-osteoclastic Raw264.7 cell line to assess the expression and subcellular localization of ANXA2 in bone cells and under HO-1 modulation. The secretome analysis of PC3 and Raw264.7 revealed uPA as a key player in the communication between osteoclast progenitors and PC3 cells. We also undertook an in-depth bioinformatics analysis to evaluate the clinical significance of ANXA2/HO-1 in PCa. We showed that high expression of either *HMOX1* or *ANXA2* correlates with longer relapse free survival time of PCa patients and that these two genes cooperate in reducing the risk of biochemical relapse. Thus, ANXA2/HO-1 rises as a critical axis in PCa.

## 2. Materials and Methods

### 2.1. Cell Lines

Human PC3 cells were obtained from the American Type Culture Collection (Manassas, VA, USA) and routinely cultured in RPMI 1640 (Invitrogen, Carlsbad, CA, USA) supplemented with 10% fetal bovine serum (FBS).

Raw264.7 is a cell line derived from murine macrophages. It can grow indefinitely as an osteoclast precursor or be stimulated to differentiate into multinucleated osteoclasts. These cells were routinely cultured in DMEM (Invitrogen, CA, USA) supplemented with 5% fetal bovine serum (FBS).

### 2.2. Hemin Pre-Treatment of PCa Cells and Co-Culture System

An in vitro bio-compartment culture system was used as a model of PCa bone metastases as previously described and slightly modified [13]. Briefly, on day 0, PC3 cells were seeded (100,000 cell/insert) in 6-well plate cell-culture inserts (0.4 mm pore; Falcon/Becton Dickinson Labware, Franklin Lakes, NJ, USA) and on day 1 they were treated with hemin (50 μM, Sigma-Aldrich, St Louis, MO, USA). Controls received fresh medium. The osteoclast precursor cells were also seeded on day 1 in 6-well tissue culture plates (300,000 cells/well). On day 2, the inserts containing the PC3 cells (pre-treated or not with hemin) were extensively washed with PBS. Then, the inserts were placed into tissue-culture plates containing the Raw264.7 cells so that the two different cell types shared the culture medium but were not in physical contact. Co-culturing of PC3 cells with pre-osteoclast was performed with DMEM plus 2% FBS for 24 h. On day 3, the cells were collected, and different parameters were analyzed. As control, each cell type (PC3 cells pre-treated or not with hemin and Raw264.7 cells) was grown alone. Cultures were done in triplicate and each experiment was assayed three times.

### 2.3. RNA Isolation, c-DNA Synthesis, and Quantitative Real-Time PCR (RT-qPCR)

Total RNA was isolated with Quick-Zol (Kalium technologies, Argentina) according to the manufacturer’s protocol. cDNAs were synthesized with RevertAid Premium First Strand cDNA Synthesis Kit (Fermentas, Waltham, MA, USA) and used for real-time PCR amplification with Taq DNA Polymerase (Invitrogen, Waltham, MA, USA) in a QuantStudio 3 Real-Time PCR System (Thermo Fisher Scientific, Waltham, MA, USA). PPIA and 36b4 were used as internal reference genes. Data obtained were analyzed using the method of 2^-ΔΔCT^ [26]. Table 1 describes the forward (Fw) and Reverse (Rv) primer sequences designed specifically for each species to avoid cross-reactivity.

### 2.4. Protein Extraction, SDS-PAGE and Western Blot (WB)

Total cell lysates and immunoblot analysis were carried out as previously described [27]. Briefly, cells were lysed with RIPA buffer (Tris HCl 50 mM pH 7.4; NaCl 150 mM; EDTA 20 mM pH 8; sodium deoxycholate 1%; SDS 0.1%; Triton X-100 1%, 1 mM Na 3VO4, 20 mM NaF and 1 mM Na4 P2 O7, pH 7.9) and homogenized. After 20 min of incubation at 4 °C, the lysates were centrifuged at 12,000 g for 20 min at 4°C and the supernatant kept at −80 °C. Lysates containing equal amounts of proteins (50 μg) were resolved on 7.5–12.5% SDS–PAGE depending on the molecular weight of the proteins under study. Page Ruler Plus Prestained Protein Ladder (Fermentas, Waltham, MA, USA) was used for the estimation of molecular weight. The proteins were blotted to a Hybond-ECL nitrocellulose membrane (GE Healthcare, Little Chalfont, UK). Membranes were blocked with 5% dry non-fat milk in TBS containing 0.1% Tween 20 (TBST) for 1h at room temperature, and incubated with primary monoclonal antibodies diluted in TBST (overnight; at 4 °C). Membranes were then washed with TBS-T (10 min; three times), and incubated with horseradish peroxidase-labelled secondary antibody (1 h at room temperature). The following antibodies were used: rabbit monoclonal anti-ANXA2 (cat. #8235; Cell Signaling; Danvers, MA, USA) diluted 1:2000, mouse anti–β-actin antibody (cat. #3700; Cell Signaling; Danvers, MA, USA) diluted 1:2000, and anti-mouse and anti-rabbit secondary antibodies (cat. #7076S and cat. #7074S, respectively. Cell Signaling, Danvers, MA, USA) diluted 1:5000.

### 2.5. Immunofluorescence Analysis

ANXA2 subcellular localization in Raw264.7 cells was evaluated by immunofluorescence (IF) staining and confocal microscopy. Microscope settings (e.g., laser intensities) were constant for all experimental conditions. For that, cells were plated over coverslip, and after co-culture experiment were rinsed with PBS and fixed in ice-cold methanol and permeabilized for 10 min with 0.5% Triton X-100/PBS, washed with PBS, and then blocked with 3% (*p/v*) BSA/PBS. Cells were incubated (overnight, at 4°C) with primary antibody rabbit monoclonal anti-ANXA2 (cat. #8235; Cell Signaling; Danvers, MA, USA) diluted 1:200 in 3% (*p/v*) BSA/PBS. Cells were then washed twice with PBS (10 min) and incubated (1 h, room temperature) with fluorescent secondary antibody Alexa Fluor 647 donkey anti-rabbit (Invitrogen, Waltham, MA, USA) diluted 1:3000 in 3% (*p/v*) BSA/PBS. Negative controls were carried out using PBS instead of primary antibodies. Cells were washed, stained with DAPI, mounted, and imaged by confocal laser scanning microscopy, which was performed with an Olympus Fluo view FV 1000 microscope, using an Olympus 60×/1.20 NA UPLAN APO water immersion objective.

### 2.6. Tissue Microarray (TMA)

The TMA was purchased from US Biomax, Inc. (Rockville, MD, USA). It included normal prostate tissue, prostate adenocarcinoma with TNM clinical stage, pathology grade, Gleason score and clinical follow-up data, benign prostatic hyperplasia (BPH), and chronic inflammation. It contained multiple cores per case to account for cellular and marker heterogeneity (including clinical data).

### 2.7. Immunohistochemical Analysis

The TMA was processed and fixed using routinely established protocols and stained as previously described [28]. Briefly, immunohistochemistry was done using the streptavidin-biotin-peroxidase complex system LSAB + kit, horseradish peroxidase (DAKO). Endogenous peroxide activity was quenched using hydrogen peroxide in distilled water (3%). Antigen retrieval was done by microwaving. Tissue slides were incubated overnight with the following primary antibodies: monoclonal mouse anti-ANXA2 (1:200) from Cell Signaling Tech, (Danvers, MA, USA) and rabbit polyclonal anti–HO-1 (1:50) from Abcam (Burlingame, CA, USA); this was followed by sequential incubations with biotinylated link antibody and peroxidase-labeled streptavidin complex. The peroxidase reaction was conducted, under microscope, using 3,3′-diaminobenzidine. Slides were counter-stained with Mayer’s hematoxylin and analyzed by standard light microscopy. Negative control slides were prepared by substituting primary antiserum with PBS. For semiquantitative analysis, the degree of staining was rated as high, moderate, low, or not detectable (3+, 2+, 1+, and 0, respectively); the staining was also observed for localization.

### 2.8. Determination of calcium ion (Ca^2+^) Concentration

Ca^2+^ concentration in the conditioned media (CM) from co-culture experiments was determined using Ca-Color AA kit (cat. #1152002; Wiener lab., Rosario, Argentina), according to the proportions indicated by the manufacturer. Briefly, co-culture experiment was carried out in DMEM medium without phenol red. Then, CM were collected and centrifuged (5 min; 1000 rpm). For the reaction, 12.5 µL of CM, or H_2_O as blank were incubated 5 min at room temperature with a mix containing Reactive A (250 µL) and Reactive B (250 µL). After this time the reaction mix from each condition was plated in a 96-well plate (3 well/condition; 150 µL/well), and read at 570 nm. Standard curve of serial dilutions to half (from 10 to 0.625 ng/dL) was carried out to calculate the Ca^2+^ concentration.

### 2.9. Secretome Analysis of Conditioned Media

To identify potential proteins involved in the interaction between PCa and bone cells, we carried out an in-depth proteomic analysis using the conditioned media (CM) obtained from the co-culture experiment of PC3 cells with Raw264.7 cells. For that purpose, CMs were collected, centrifuged (2000 rpm, 10 min), and concentrated by centrifugation (3 × 15 min, 6500 g) in VivaSpin® tubes (vivaspin® 20 Polyethersulfone; cut off > 10 kDa; #VS2001; Sartorius, Germany). For the protein identification we performed LC ESI-MS/MS and proceeded as described in Paez et al. [16]. The obtained peptides were compared against human and murine protein databases to obtain the list of proteins in each sample. We then compared the lists to obtain the “differential proteins” in the co-culture of PC3 with Raw264.7 or in the co-culture of hemin pre-treated PC3 with Raw264.7 (against the list of proteins form the CMs of PC3 and Raw264.7 cells growing alone).

### 2.10. Enzyme-Linked Immunosorbent Assay (ELISA)

PC3 cells were treated or not with hemin (50 μM; 24 h). CMs of the treated and untreated cells was collected and concentrated by centrifugation (3 × 15 min, 6500× *g*) using the VivaSpin® tubes (Sartorius, Göttingen, Germany). To measure the levels of uPA released in the CMs, we used a 1:10,000 dilution (determined as the optimal working dilution after performing a calibration curve) on a commercial Human ELISA Kit (Abcam, Cambridge, MA, USA), and proceeded according to the manufacturer’s instructions. uPA concentration was the determined using a GloMax luminometer (Promega, Madison, WI, USA) by absorbance at 450 nm, 30 min post enzymatic reaction.

### 2.11. Statistical Analysis

All results are shown as mean ± SD of three separate independent experiments unless stated otherwise. ANOVA with Tukey post-test was used to ascertain statistical significance with a threshold of *p* <0.05 (*), *p* < 0.01 (**), and *p* < 0.001 (***).

### 2.12. Bioinformatics Analysis

#### 2.12.1. Information Source and Eligibility Criteria (Oncomine)

We searched the public cancer microarray database Oncomine (http://www.oncomine.org) to identify expression microarray data sets that compared gene expression in prostate adenocarcinoma versus normal prostate gland. To be included in our study, a data set was required to (1) be generated from human tumors, (2) compare prostate adenocarcinoma versus normal prostate gland, (3) have a *p*-value < 0.05, and (4) have a fold change > 1.5 and/or have a gene rank within the top 10%. 

Search/study selection: we performed a search for HMOX1 or ANXA2 as the search term. The resulting studies were analyzed on the basis of healthy prostate gland versus prostate adenocarcinoma. Cited literature was reviewed to confirm that the analysis was as documented in the Oncomine database.

#### 2.12.2. Information Source and Eligibility Criteria (The Cancer Genome Atlas (TCGA) and Fred Huchinson CRC (GSE74685)

To be included in our study on HMOX1 and ANXA2 expression, the data sets were required to (1) include gene expression data from PCa patients’ samples, (2) the study consisted of ≥ 60 samples, and (3) the study was published and available in public repositories.

Search/study selection: we used (1) the dataset from the Prostate Adenocarcinoma Project of The Cancer Genome Atlas (TCGA-PRAD) (http://cancergenome.nih.gov/) that has RNAseq data from 497 prostate tumor samples and normal adjacent tissue, measured by massively parallel sequencing (lluminaHiSeq); (2) the dataset from the Nelson Lab, Fred Huchinson CRC (GSE74685) [29], a prostate cancer patient’s cohort comprised of 171 samples from primary (*n* = 22) or metastatic (*n* = 149) tumors from 63 PCa patients, with complete Agilent 44K whole human genome expression oligonucleotide microarray.

Statistical analysis: we used GraphPad Prism software (La Jolla, CA, USA). Student’s *t*-test was used for testing differences in gene expression across tissue samples. *p*-values less than 0.05 were considered statistically significant.

#### 2.12.3. Correlation of HMOX1 and ANXA2 Expression with Relapse-Free Survival

Information source and eligibility criteria (GEO: Gene Expression Omnibus): to study the impact of HMOX1 and/or ANXA2 expression levels on relapse-free survival (RFS) of PCa patients, two data sets were selected according to the following criteria: (1) the study included metadata for each patient, with ≥ 5 years of follow-up overall or relapse-free survival, (2) the study consisted of ≥ 60 samples, and (3) the study was published and available on GEO (Gene Expression Omnibus).

Search/study selection: we used the dataset from Ross-Adams 2015 (GSE70770) [30] GPL10558 series, a prostate cancer patient’s cohort with 206 samples from men with prostate cancer undergoing radical prostatectomy and clinical follow-up at 8 years, including relapse information (biochemical recurrence was defined according to European Guidelines as a persistent rise above 0.2 ng/mL). Tumor sample expression of 31,000 transcripts was measured by 47,000 probes using the Illumina HumanHT-12 V4.0 platform.

Statistical analysis: Stata software (StataCorp LLC, College Station, TX, USA) was used to explore the patient’s relapse-free survival and to generate Kaplan–Meier curves. To find the cutoff point to stratify patients in two groups based on the gene expression levels, we used the minimal *p*-value approach from the Cutoff Finder tool [31]. For univariable and multivariable analyses of prognostic factors, Log-rank test and Cox proportional hazard model regression was employed. Statistical significance was set at *p* < 0.05. Correlation analysis: Pearson correlation coefficient was used to measure the linear correlation between the expression of HMOX1 and ANXA2 within each dataset.

## 3. Results

### 3.1. Analysis of ANXA2 Expression in PC3 and Bone Progenitor Cells Grown in Co-Culture Conditions. Effect of HO-1 Induction

**Figure 1:**
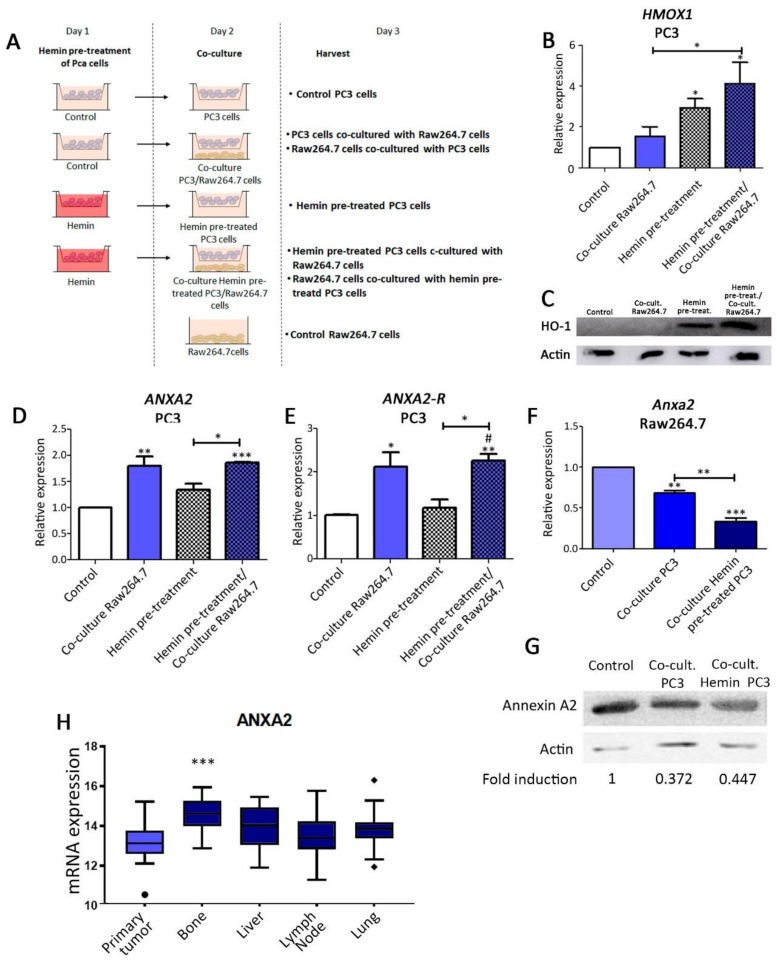
Analysis of annexin 2 (*ANXA2*) expression in PC3 and bone progenitors’ cells grown in co-culture conditions. Effect of heme oxygenase 1 (HO-1) induction. (**A**) Schematic representation of the co-culture transwell system of PC3 and Raw264.7 cells. Expression levels of (**B**,**C**) HO-1 assessed by real-time PCR (RT-qPCR) and WB; (**D**) *ANXA2* and (**E**) *ANXA2-R* assessed by RT-qPCR in PC3 cells pre-treated or not with hemin (50 μM; 24 h), grown alone or in co-culture with Raw264.7 cells. The values were relativized using *PPIA* as a reference gene and normalized to controls. Expression levels of *Anxa2* assessed by RT-qPCR (**F**) (relativized to 36b4 as a reference gene) or Western blot (**G**) (relativized to β-actin, as a reference protein), in Raw264.7 cells grown alone or in co-culture with PC3 cells pre-treated or not with hemin (50 μM; 24 h). (**H**) Expression levels for *ANXA2* in primary tumor vs. different sites of metastasis (bone, liver, lymph node, lung), analyzed on the GSE74685 dataset (*n* = 171). Significant differences: * *p* < 0.05; ** *p* < 0.01; *** *p* < 0.001.

HO-1 has the potential to modify the bone microenvironment impacting on PCa bone metastasis [13,14,32]. We have previously described the HO-1 interactome in PCa through a proteomics approach, identifying HO-1 molecular partners implicated in cell adhesion and cell–cell communication [16]. One of these molecular partners was ANXA2. As mentioned above, the ANXA2/ANXA2-R is closely related to the bone compartment [22]. Our first approach was to investigate the effect of soluble factors produced by co-culturing PC3 cells (PC3) and pre-osteoclastic progenitor cells (Raw264.7) on the ANXA2/ANXA2-R axis. PC3 cells, known to display osteolytic properties when growing in bone [33], were pre-treated or not with the pharmacological inducer of HO-1, hemin (50 µM; 24 h), and then cultured for 24h with Raw264.7 cells in a transwell system. Hemin, is the substrate of HO-1, also known as a physiological inducer of HO-1, that increases both HO-1 mRNA and protein levels [34]. Both, PC3 and bone progenitor cells shared the medium but were not in direct physical contact (Figure 1A). First, HO-1 induction by hemin in PC3 cells, growing alone or in co-culture with Raw264.7cells, was confirmed by RT-qPCR (Figure 1B) and Western Blot (WB) (Figure 1C). Next, *ANXA2* and *ANXA2-R* transcription levels were assessed showing a significant increase in PC3 cells co-cultured with pre-osteoclastic cells as determined by RT-qPCR (Figure 1D,E). No effect was detected by hemin pre-treatment (Figure 1D,E). Surprisingly, *Anxa2* mRNA levels were significantly decreased in Raw264.7 cells after co-culture with PC3 cells, and this downregulation was even more pronounced when PC3 cells were pre-treated with hemin (Figure 1F). This downregulation was also confirmed at protein levels by Western blot (Figure 1G). These in vitro results provide evidence that the interaction of PC3 cells with bone progenitor cells modulate ANXA2 expression in both PC3 and bone cells. This modulation may favor tumor cell colonization in the bone metastatic compartment.

To investigate whether the co-culture system had an effect on cell invasion, PC3 cells pre-treated or not with hemin were plated on Matrigel in a transwell insert and incubated alone or in co-culture with Raw264.7 cells. Results show that the co-culture condition significantly increased the invasiveness of PC3 cell and in accordance with our previous published results, hemin pre-treatment of PC3 cells (growing alone or in co-culture) significantly reduced the invasive capacity of tumor cells (Supplementary Figure 1A,B). These results point to soluble factors released by Raw264.7 cells that may act as chemoattractant on PC3 cells, modulating their invasive capacity. To further explore other parameters involved in colonization of the metastatic niche, we also assessed the effect of the CM obtained from the transwell co-culture systems (PC3 and Raw264.7), on tumoral cell protrusions. PC3 cells pre-treated or not with hemin were co-cultured with or without Raw264.7 cells. CM from the different experimental protocols was then added to PC3 cells (Supplementary Figure 2A). Cell contact density and number of protrusions were evaluated by confocal microscopy (Supplementary Figure 2B). No alterations were observed for contact density and cell–cell distance for cells cultured with the CM of PC3 cell growing alone or pre-treated with hemin (Supplementary Figure 2C). However, CM from the co-culture system reduced the number of contacts among PCa cells (Supplementary Figure 2C; PC3 + CM2 vs. PC3 + CM1) and hemin pre-treatment prevented this fall (Supplementary Figure 2C; PC3 + CM4 vs. PC3 + CM2). Intriguingly, the CM from co-culture systems impacted negatively on the membrane filopodia density of PC3 cells (Supplementary Figure 2C; PC3 + CM2 vs. PC3 + CM1). These effects were prevented by hemin-pre-treatment (Supplementary Figure 2C; PC3 + CM4 vs. PC3 + CM2). Together, these findings suggest that induced HO-1 expression in PC3 cells alters the soluble factors released to the CM in the co-culture systems, which in turn affect cell filopodia and cell adhesion zippering among cells. A more adhesive phenotype of tumoral cells may prevent them from extravasation and homing to other organs.

### 3.2. ANXA2 Transcriptomic Levels Increased in Metastatic Tumors Compared with Primary Human PCa

In parallel, we assessed *ANXA2* expression at the transcriptomic levels in human samples of primary and metastatic PCa using the dataset reported by Kumar et al. (GSE74685) [29] which involved 149 samples of metastasis (20 bone metastasis) and 22 primary tumor samples derived from 63 PCa patients. The analysis showed that *ANXA2* expression was significantly higher in the bone metastatic samples when compared with the primary tumor site (*p* < 0.001), and no significant difference was detected for the other PCa metastatic sites (lung, liver, and lymph node) (Figure 1H). These results are in accordance with our in vitro results, where *ANXA2* expression is increased when co-cultured with the bone progenitor cells.

### 3.3. ANXA2 Subcellular Localization in Osteoclast Progenitors is Altered When Co-Cultured with Tumor Cells and under HO-1 Induction

**Figure 2:**
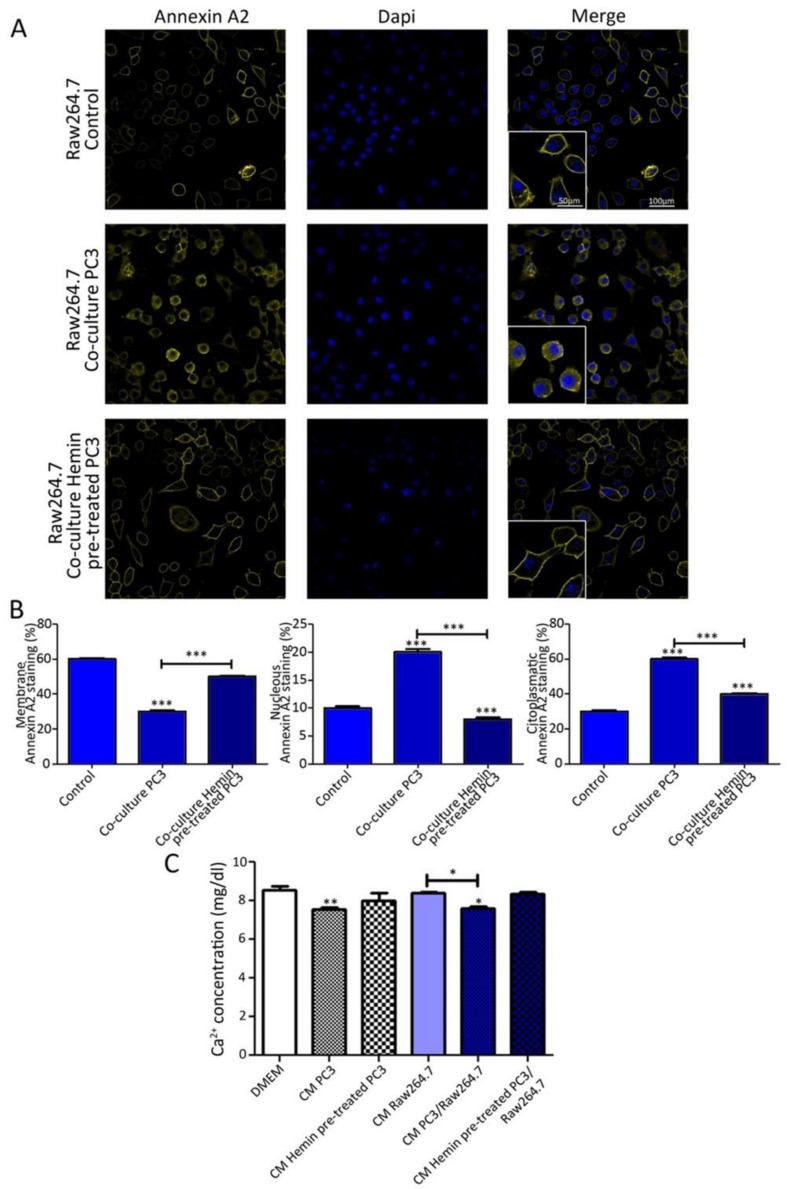
ANXA2 subcellular localization in osteoclasts progenitors when co-cultured with tumor cells and under HO-1 induction. (**A**) Immunofluorescence (IF) staining and confocal microscopy analysis for ANXA2 on Raw264.7 grown alone or in co-culture with PC3 cells pre-treated or not with hemin (50 μM; 24 h). (**B**) Semi-quantitative analyses of the IF by segmentation of the whole cell, intracellular, and nucleus (DAPI). The florescence intensity for ANXA2 was calculated using ImageJ and normalized to cell size (*n* ≥ 180 cells for each condition). Percentage of staining in each compartment by cell-by-cell analysis is depicted. (**C**) Ca^2+^ concentration levels of conditioned media from PC3 and Raw264.7 cells growing in co-culture conditions. Statistical significance is shown with respect to the unconditioned DMEM medium. Bars depict comparisons among the other conditions. Significant differences: * *p* < 0.05, ** *p* < 0.01; *** *p* < 0.001.

To get a deeper understanding into the effect of the co-culture in the modulation of ANXA2, we evaluated the subcellular localization of this protein in both cell lines. By confocal microscopy, we showed that ANXA2 was mainly localized in the cell membrane of Raw264.7 when cultured alone (Figure 2A upper panels and B), while intracellular immunofluorescence was detected when bone progenitor cells were co-cultured with PC3 cells (Figure 2A middle panels and B). Interestingly, hemin pre-treatment of PC3 cells partially prevented the ANXA2 intracellular relocation provoked by the co-culture in Raw264.7 cells (Figure 2A lower panels and B). However, in line with all the published literature regarding the role of ANXA2 in cell adhesion [22,35,36], the rounding up of cells observed could further reflect the internalization of ANXA2 as observed from this experiment. Thus, less ANXA2 in the cell membrane is in accordance with a decreased attachment of cells to the substrate.

No significant differences were observed under the same experimental conditions for ANXA2 localization in PC3, stating that neither the co-culture nor the hemin pretreatment affects ANXA2 cellular localization in this cell line (data not shown).

Although the mechanism of action of ANXA2 is poorly described, this protein is able to localize to the cell membrane forming an heterotetramer with the S100A10 (P11) protein and this process depends on Ca^2+^ availability [35,37]. Thus, we next assessed the levels of this ion in the conditioned medium (CM) of the co-culture. A significant reduction in Ca^2+^ levels was observed when bone progenitor cells were co-cultured with PC3 cells compared with the CM of Raw264.7 growing alone (*p* <0.05) (Figure 2C). Further, hemin pre-treatment of PC3 cells impaired the reduction in Ca^2+^ concentration in the CM triggered by the co-culture (Figure 2C). Thus, the reduced Ca^2+^ concentration in the CM, as a consequence of the presence of tumor cells in the co-culture, is consistent with the internalization of ANXA2 from the plasma membrane to the cytoplasm in the osteoclastic precursors (Figure 2A,B).

### 3.4. Secretome Analysis Reveals uPA as a Key Player in the Communication between Osteoclast Progenitors and PC3 Cells

Our results prompted us to evaluate the possible mechanism by which ANXA2 could be internalizing from the cell membrane to the cytosolic compartment in the osteoclastic precursors upon co-culture with PC3 cells, and how these effects were reversed when Raw246.7 cells were co-cultured with PC3 cells pre-treated with hemin.

**Figure 3:**
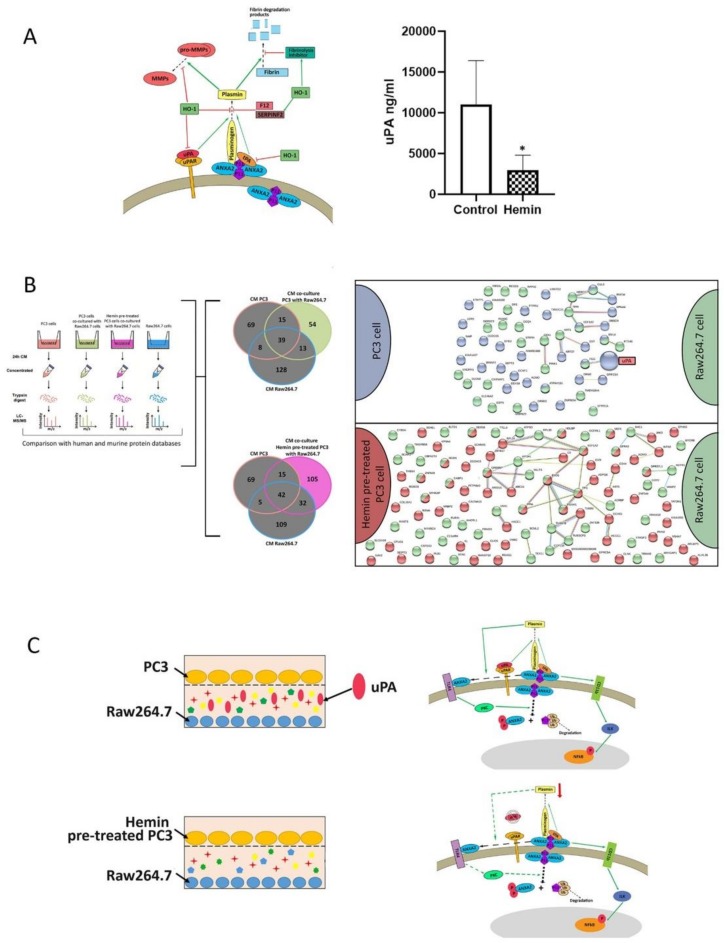
Secretome analyses of conditioned media (CM) from the co-culture transwell system between PC3 and Raw246.t cells. Effect of HO-1 induction. (**A**) Model of plasmin regulation by cell surface annexin A2 and HO-1. Annexin A2 forms a heterotetrameric complex, consisting of two molecules of annexin A2 and one copy of the S100A10P11 (P11) dimer, which binds to the tissue-plasminogen activator tPA and plasminogen. The urokinase-plasminogen activator (uPA) is bound to its receptor (uPAR) and forms the uPA/uPAR complex that co-localizes with the annexin A2-S100A10 complex. This causes plasminogen to cleave into plasmin, which activates pro-MMPs (matrix metallo-proteases) into active MMPs and also degrades fibrin. High expression of HO-1 decreases the extracellular matrix degradation by inhibiting the uPA/uPAR complex and inactivating pro-MMPs. It can also inhibit the cleavage of plasminogen into plasmin, directly or by activating SERPINF2, which together with F12 inactivates plasmin formation. Further, high HO-1 expression causes activation of fibrinolysis inhibitors, inhibiting fibrin degradation (left panel). uPA concentration, expressed as ng/mL, in conditioned media of PC3 cells treated with hemin (50 µM, 24 h) or PBS as control. One of three independent experiments is shown (*p < 0.05) (right panel). (**B**) Schematic workflow of sample obtaining and processing of conditioned media (CM) for analysis by mass spectrometry. Exclusion/inclusion criteria to form differential protein lists represented as Venn diagrams, where the areas colored with green or pink correspond to the differential proteins of CMs from the co-culture of PC3 with Raw264.7 or Hemin pre-treated PC3 with Raw264.7, respectively. Gray areas correspond to shared proteins, excluded from the differential lists. Protein–protein interactions between the differential proteins of CMs from the co-culture of PC3 with Raw264.7 or Hemin pre-treated PC3 with Raw264.7. (**C**) Schematic representation of the proposed model for proteins associated with prostate cancer (PCa) and bone metastasis affected by the interaction of soluble factors between PC3 and Raw264.7 cells.

We had previously demonstrated a strong association between HO-1 and the urokinase-plasminogen activator (uPA) and its receptor (uPAR) axis in PCa. This pathway, through the activation of the plasminogen system, degrades ECM, and consequently modulates tumor cell membrane protrusion, invasion, and motility [38]. Our results unveiled that when PCa cells overexpressed HO-1 there was a direct transcriptional regulation of the critical players in the uPA/uPAR cascade, such as the downregulation of the axis activators: uPA/uPAR (PLAU/PLAUR) and tPA (PLAT), and the upregulation of the axis inhibitors: thrombin activator of fibrinolysis inhibitor (CPB2), alpha 2 anti-plasmin, (SERPINF2) and Factor XIIA (F12) (Figure 3A, left panel) [16]. Therefore, we decided to evaluate whether the impairment of the uPA/uPAR axis was accompanied by a decrease in the levels of this urokinase in the extracellular environment. For this purpose, we performed an enzyme-linked immunosorbent assay (ELISA) on conditioned media of PC3 cells collected after hemin treatment. Results showed a significant decrease in uPA concentration under HO-1 induction compared with control (Figure 3A, right panel).

At the endothelial cell surface, ANXA2⋅P11 tetramer acts as a coreceptor for plasminogen and tissue plasminogen activator (tPA), accelerating tPA-dependent activation of the fibrinolytic protease, plasmin, the enzyme that is responsible for thrombus dissolution and the degradation of fibrin [39]. Plasmin activates pro-MMPs (matrix metallo-proteases) into active MMPs and further activates pro-uPA into active uPA [39].

In the presence of uPA, plasmin increases, activating ANXA2 phosphorylation, disassembling the ANXA2⋅P11 tetramer allowing the ubiquitination and degradation of P11 and the reduced surface level of ANXA2 [40]. This mechanism is described for the macrophage lineage.

Thus, we assessed if uPA was present in the secretome of the co-culture CMs. We performed an in-depth proteomic analysis (LC ESI-MS/MS) to evaluate the soluble factors released to the CM by both PC3 and RAW264.7 cells and assessed the differential proteins from the co-culture CMs when PC3 cells were pre-treated or not with hemin, comparing against the CM from both cell lines grown alone. To analyze these proteins, we proceeded as described in the data analysis pipeline (Figure 3B, left panel). The proteomic yield obtained was 54 differential human and murine proteins for the PC3 and Raw264.7 co-culture CM and 105 differential human and murine proteins for the hemin pre-treated PC3 and Raw264.7 co-culture CM. In line with our previous results, uPA was present in the co-culture CM and absent in the co-culture CM when PC3 was pre-treated with hemin (Figure 3B, right panel). The query against the human protein databases evidenced that uPA was a human protein and hence was released by the tumor cells (Supplementary Tables S1 and S2). These results together with the observations that when HO-1 is induced in tumor cells there is a downregulation of the uPA/UPAR pathway resulting in a halted release of uPA, further support the shift in ANXA2 IF staining observed in Figure 2. The schematic representation observed in Figure 3C shows that when uPA is released to the CM in the co-culture conditions it triggers the dissemble of the ANXA2⋅P11 tetramer, allowing ANXA2 to internalize and increase its presence in the cytosolic and nuclear compartments. PC3 cells overexpressing HO-1 growing in co-culture with the osteoclastic precursors, appear to reverse this effect. Of note, no ANXA2 was found in the co-culture secretome suggesting that ANXA2 was not released to the CM.

### 3.5. Clinical Relevance of ANXA2 and HMOX1 in PCa

**Figure 4:**
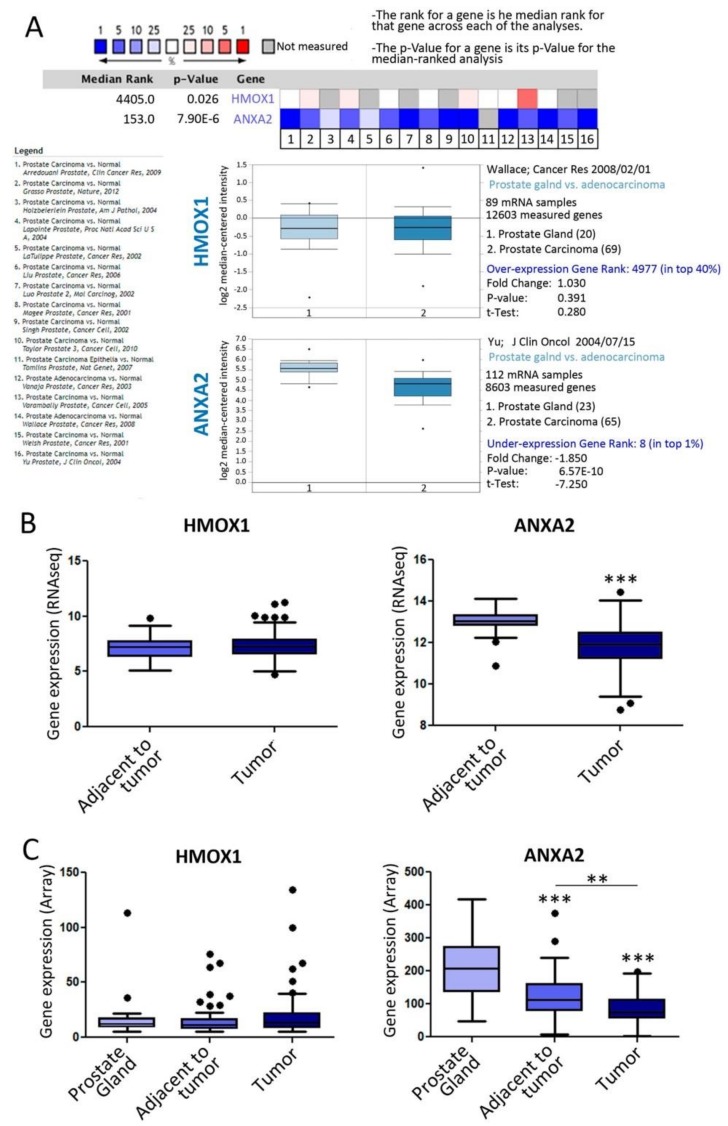
Clinical relevance of *ANXA2* and *HMOX1* in PCa. (**A**) Meta-analysis of multiple microarray datasets for *HMOX1* and *ANXA2* expression (Oncomine). One representative box plot is shown for each gene comparing its expression profile between group 1: Prostate Gland and group 2: Prostate Adenocarcinoma (*n* = 1128). (**B**) *HMOX1* and *ANXA2* expression levels in Tumor and Adjacent to tumor human samples on TCGA-PRAD RNAseq dataset (*n* = 497). (**C**) *HMOX1* and *ANXA2* expression levels in normal prostate gland, adjacent to tumor, and tumor samples on the GSE6919 microarray dataset (*n* = 171). Significant differences: ** *p* < 0.01, *** *p* < 0.001.

To further assess the clinical significance of these molecules we performed a bioinformatics analysis using public databases repositories. First, we searched the public cancer microarray database, Oncomine (http://www.oncomine.org) (Figure 4A). We found significantly lower levels of *ANXA2* expression in prostate adenocarcinoma compared with normal prostate gland (Median Rank: 153; *p* = 7.9× 10^−6^. *n* = 973 patient samples) (Figure 4A). The meta-analysis combining data from the independent data sets assessed, showed that *ANXA2* lies within the 1%–16% of the most consistently underexpressed genes for prostate adenocarcinoma versus normal prostate gland. *HMOX1* presented no significant variation for the same comparison (Figure 4A). Second, we examined the expression of both genes in human patient samples using the data from Prostate Adenocarcinoma Project of The Cancer Genome Atlas (TCGA-PRAD, *n* = 497) [41]. Accordingly, a significant reduction in *ANXA2* expression was detected in the tumor samples compared with normal adjacent tissue (*p* < 0.001) (Figure 4B). Once again, no significant variation was observed in *HMOX1* mRNA levels for the same comparison (Figure 4B). Third, we assessed the expression of these genes in the GSE6919 dataset, comprising normal, adjacent, and primary tumor human tissue samples (*n* = 171). Accordingly, *ANXA2* expression was significantly downregulated in tumor vs. normal tissue (*p* < 0.0001) (Figure 4C) and in tumor vs. normal adjacent (*p* < 0.0001) (Figure 4C).

**Figure 5:**
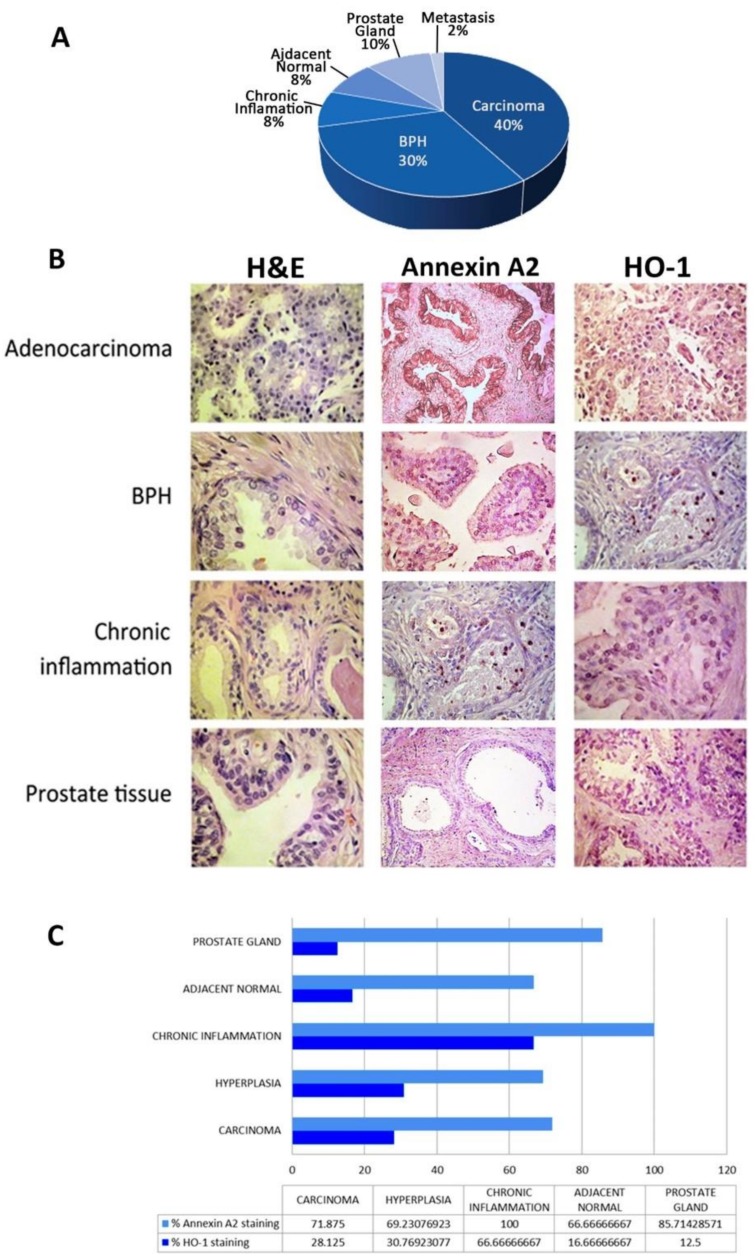
Immunohistochemical (IHC) assessment of HO-1 and ANXA2 in a human tissue micro arrays (TMA). Prostate disease spectrum tissue microarray was used for the IHC analyses, containing 32 cases of adenocarcinoma, two metastasis, 26 hyperplasia, six chronic inflammation, six cancer adjacent prostate tissue, and eight normal tissue. (**A**) Pie chart shows the distribution of the disease stages analyzed in the TMA. (**B**) Representative images for adenocarcinoma, BPH, chronic inflammation, and normal gland stained with H&E or immunoassayed for Annexin 2 or HO-1. (**C**) Graph represents the percentage of positive samples for ANXA2 or HO-1 immunostaining across the different prostate tissue samples.

We then used tissue microarrays (TMAs) to further assess the significance of HO-1 and ANXA2 in human PCa. We selected a commercial TMA (US Biomax Inc # pR8011a) that comprised metastatic prostate tissue, adenocarcinoma of different stages, BPH, chronic inflammation, and normal prostate (Figure 5A). Our analysis showed that HO-1 staining was heterogeneous, and some samples presented positive immunoreactivity both in the nucleus and in the cytoplasm (Figure 5B, middle panels). Regarding ANXA2 immunohistochemistry results, it is important to highlight that when there was positive immunoreactivity it was mostly seen in the cytosolic compartment of human tumor samples (Figure 5B, right panels). Concerning HO-1 staining across the different prostate lesions, it is clear that chronic inflammation displayed the highest number of positive cases (Figure 5C). For ANXA2 there is a reduction in the positive staining between normal prostate and carcinoma (Figure 5C). In the case of prostate adenocarcinoma there was 53% of samples HO-1(-)/ANXA2(+), 18.7% HO-1(+)/ANXA2(+), 9.6% HO-1(+)/ANXA2(-), and 18.7% HO-1(-)/ANXA2(-) (Figure 5C).

### 3.6. Analysis of HMOX1 and ANXA2 as Risk Predictors of Clinical Outcome in PCa

**Figure 6:**
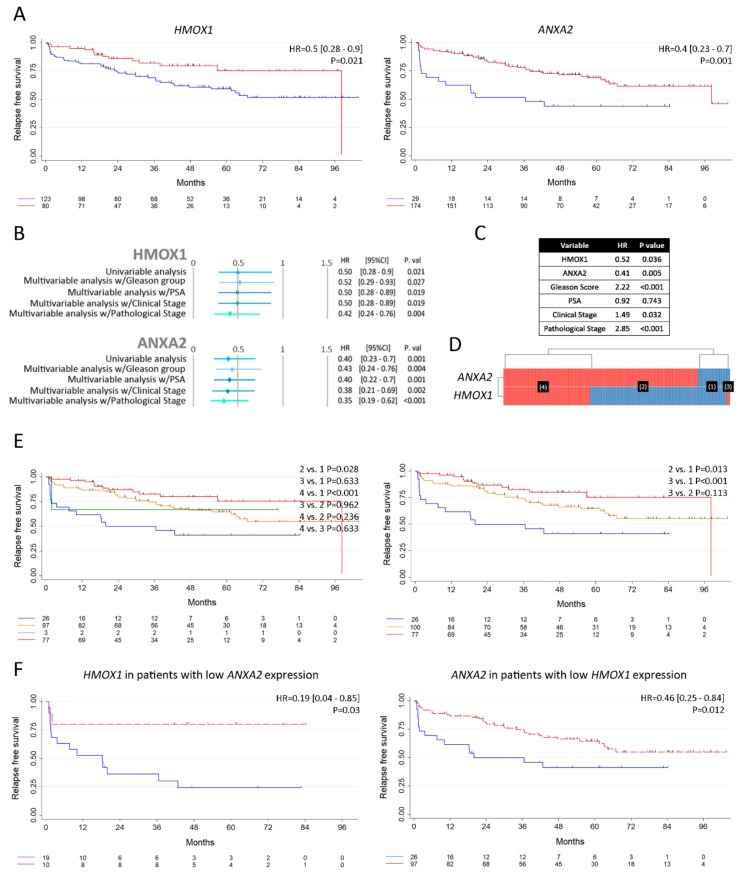
Analysis of *HMOX1* and *ANXA2* as risk predictors of clinical outcome in PCa. (**A**) Kaplan–Meier (KM) curves for relapse free survival (RFS) (months) and risk table for PCa patients with high (red) or low (blue) expression of *HMOX1* and *ANXA2* in *Ross-Adams* dataset (*n* = 206). Low expression group was taken as a reference group. Logrank P, HR: Hazard ratio [95% confidence interval] and Cox P are specified. (**B**) Univariable and multivariable analyses are presented by forest plots for relapse free survival regarding *HMOX1* (upper panel) or *ANXA2* (lower panel) expression. Multivariable analysis w/Gleason group = adjusted for Gleason groups (6; 7 (3+4); 7 (4+3); 8-10). Multivariable analysis w/PSA = adjusted for PSA serum levels at diagnosis (PSA groups (ng/mL): < 4; 4-10; > 10). Multivariable analysis w/clinical stage = adjusted for patients’ clinical stage. Multivariable analysis w/pathological stage = adjusted for patients’ pathological stage. w/= with. (**C**) Multivariable analyses based on the mRNA expression of *HMOX*, *ANXA*2, Gleason Score, PSA at time of diagnosis, clinical and pathological stage for patients with PCa in the *Ross-Adams* dataset. (**D**) Heatmap depicting low (blue) or high (red) *HMOX1* and *ANXA2* mRNA expression for patients with PCa according to the *Ross-Adams* dataset. Patient subgroups are presented in black boxes. (E) RFS of patients with low *HMOX1* and *ANXA2* expression (*n* = 26) (1), high *ANXA2* and low *HMOX1* expression (*n* = 97) (2), low *ANXA2* and high *HMOX1* expression (*n* = 3) (3), and high *ANXA2* and *HMOX1* expression (*n* = 78) (4) (left panel); RFS of patients with low *HMOX1* and *ANXA2* expression (*n* = 26) (1), either high *ANXA2* or *HMOX1* expression (*n* = 100) (2), and high *ANXA2* and *HMOX1* expression (*n* = 78) (3) (right panel). (F) KM curves for RFS and risk table for PCa patients with high (red) or low (blue) expression of *HMOX1*, segregated based on low *ANXA2* (left panel); and high (red) or low (blue) expression of *ANXA2*, segregated based on low *HMOX1* (right panel). Stata software (StataCorp LLC, Texas, USA) was used to assess the patient’s overall and RFS and to generate Kaplan–Meier curves. Minimal *p*-value approach from the Cutoff Finder tool [31] was used to stratify patients in two groups based on the gene expression levels. For univariable and multivariable analyses of prognostic factors, Log-rank test and Cox proportional hazard model regression was employed. Statistical significance was set at *p* < 0.05.

Next, we assessed the association of the *HMOX1* and *ANXA2* with the prognosis of PCa. We evaluated the risk of relapse in PCa patients that had undergone radical prostatectomy. We used the *Ross-Adams* dataset (GSE70770) [30] (*n* = 206) and assessed the relapse-free survival (RFS) for these patients. Results show that higher expression levels for both *HMOX1* and *ANXA2* are associated with better RFS in PCa patients (HR: 0.5, *p* = 0.021 for *HMOX1*; HR: 0.4, *p* = 0.001 for *ANXA2*) (Figure 6A, left and right panel, respectively).

To validate the potentiality of both genes to increase the RFS of PCa patients, multivariable analyses were performed in the presence of significant clinico-pathological parameters previously associated with increased death risk. These parameters included Gleason score (GS), PSA levels at time of diagnosis, and clinical and pathological stage (Figure 6B). The forest plots show that high expressions of *HMOX1* and *ANXA2* are significantly correlated with higher RFS and behave independently from the patient’s GS, PSA levels, and clinical and pathological stage (Figure 6B).

In order to assess variable independence, we adjusted the model to include simultaneously *HMOX1, ANXA2*, GS, PSA levels at time of diagnosis and clinical and pathological stage. Results showed that *HMOX1*, *ANXA2*, GS, clinical and pathological stage behave independently when predicting death risk (Figure 6C). Next, we categorized PCa patients based on *HMOX1* and *ANXA2* gene expression levels. The heatmap in Figure 6D depicts patient subgroups with low *HMOX1* and *ANXA2* expression (*n* = 26) (group 1), high *ANXA2* and low *HMOX1* expression (*n* = 97) (group 2), low *ANXA2* and high *HMOX1* expression (*n* = 3) (group 3), and high *ANXA2* and *HMOX1* expression (*n* = 78) (group 4) (Figure 6D). Next, we performed Kaplan–Meier (KM) survival curves to evaluate the RFS of these patient subgroups. Patients in groups 2 and 4 had significantly increased RFS compared with patients in group 1 (Figure 6E, left panel). Since group 3 consisted of very few patients, we merged it with group 2, creating a new group of patients with either high expression of *HMOX1* or *ANXA2* for further analysis and named it group 2. Figure 6E, right panel, shows the RFS of patients categorized based on these three new groups. Patients with either high expression of *HMOX1* or *ANXA2* (group 2) showed improved RFS compared with patients with low expression for both genes (*p* = 0.013) (Figure 6E, right panel).

These results are further evidenced in the KM curves presented in Figure 6F, where within the subset of patients with low expression for *ANXA2*, those with higher *HMOX1* expression showed improved RFS compared with patients with low *HMOX1* expression (HR: 0.19, *p* = 0.03) (Figure 6F, left panel). Similar results were found for *ANXA2* expression levels analyzed in the subset of patients with low *HMOX1* expression, where increased *ANXA2* significantly improved the RFS compared with patients with low *ANXA2* levels (HR: 0.46, *p* = 0.012) (Figure 6F, right panel). To summarize, high expression of either *HMOX1* or *ANXA2* correlates with longer RFS in PCa patients, and these two genes cooperate in reducing risk of relapse.

## 4. Discussion

Taking into account the involvement of HO-1 in PCa demonstrated by us and other authors [11,16,42,43], we were keen to identify HO-1 molecular partners responsible for the biological effects provoked by HO-1 induction in prostate tumorigenesis. The literature reflects controversial results regarding the role of HO-1 in carcinogenesis [7]. Some evidence suggests that HO-1 can act as a protective enzyme, decreasing the risk of developing certain tumors. However, much more was reported about its pro-tumoral function. The pro-carcinogenic effects of HO-1 are associated with its cytoprotective [44] and antiapoptotic activity [45], which results in increased survival of tumor cells and resistance to therapies. In addition, HO-1 can act as a pro-angiogenic mediator which favors tumor vascularization [46] increasing the metastatic potential. On the other hand, there are numerous studies that show that the anti-inflammatory action of HO-1 can result in immunosuppression, favoring tumor progression [9]. Although it may seem that HO-1 facilitates tumor growth and metastasis, the effects depend on the type of cancer [8,47,48]. HO-1 was shown to have anti-tumor activities in breast cancer [49] as well as in non-small cell lung cancer [50] and colorectal cancer [51]. However, these same authors reported the pro-tumor action of the same protein in astrocytoma, glioma, and head and neck squamous cell carcinomas [52,53]. As previously mentioned, our work showcased the nuclear expression of HO-1 in human treatment naive prostate carcinomas and in benign BPH samples [42]. In addition, we demonstrated that the pharmacological and genetic induction of HO-1 triggered its nuclear translocation and inhibited proliferation, migration, and invasion in vitro, and decreased tumor growth in vivo [11].

Given that our previous proteomics analysis depicting HO-1 interactome in PCa unveiled ANXA2 among the HO-1 interactor proteins [16], in this work, we sought to analyze the relevance of ANXA2/HO-1 in PCa and bone metastasis. Of note, we had previously published that HO-1 is involved in the bone physiology [14] and participates in PCa bone metastasis [13,14]. Although, men with PCa display in most cases osteoblastic metastases [54], an underlying osteoclastic component should not be overlooked [25]. To explore the contribution of HO-1 in the communication between prostate tumor cells and bone precursor cells, we used an indirect co-culture system between PC3 and Raw264.7 cell lines. Our results showed a significant increase of *ANXA2* and *ANXA2-R* expression in PCa cells grown in the presence of osteoclasts precursors. We also found that ANXA2 undergoes re-localization from the cell membrane to the cytoplasmic compartment of the bone precursor cells, when Raw264.7 cells were co-cultured with PC3 cells. This effect could be explained, at least in part, by the decrease in Ca^2+^ concentration detected in the CM. According to the literature and as previously described, ANXA2 is located in the cell membrane forming a heterotetramer with the S100A10 (P11) protein and this process is dependent on the Ca2+ availability [55]. Neither the change in ANXA2 localization nor the decrease in Ca^2+^ levels were detected when PC3 cells had undergone HO-1 induction before the co-culture with the bone progenitor cells.

ANXA2 is an autocrine/paracrine factor that stimulates osteoclasts formation and bone resorption [56]. In tumor cells ANXA2 regulates adhesion, invasion, proliferation, and migration [57]. Thus, ANXA2 plays a key role in establishing PCa bone metastasis [22,35], facilitating the tumor cells homing in the bone marrow [22]. Literature evidences that ANXA2/ANXA2R exerts an important role both in the bone physiology and in the growth of tumor cells in the skeletal tissue [56].

Thus, the expression and/or localization of ANXA2 could be driving the tumor cells to the bone niche favoring their homing to this second site and, in turn, promoting the activation of osteoclasts, altering bone homeostasis. HO-1 appears to interfere with that signaling.

When performing invasion assays results show that the co-culture condition significantly increased the invasiveness of PC3 cell and in accordance with our previous published results, hemin pre-treatment of PC3 cells (growing alone or in co-culture) significantly reduced the invasive capacity of tumor cells. Indeed, when analyzing other parameters involved in the colonization of the metastatic niche, such as tumor cell adhesion, zippering, and protrusions, results showed that PC3 cells exposed to CM from the co-culture system, displayed reduced membrane filopodia density and contact among cells, effects reverted by hemin pre-treatment. Retraction of cell protrusions could be a sign of rounding up of cells and may be one of the characteristic features of cell detachment. Our results suggest that HO-1 forced-expression prevents PCa cells from extravasation to other homing organs. Altogether, our results evidence the importance to the soluble factors released by both tumor and bone progenitor cells acting, favoring the colonization of the bone niche.

Herein, we report that microarray and RNAseq data from patients’ samples available at public repositories, show *ANXA2* to be significantly downregulated in PCa compared with normal prostate. Furthermore, when performing a meta-analysis through the Oncomine platform, this gene appeared among the 10% of the most consistently downregulated genes in PCa compared with normal gland, reflecting its importance in prostate tumorigenesis. We were able to support these results in our laboratory by immunohistochemical analysis using a commercial TMA, which contained samples of different prostate pathologies. In addition, HO-1 levels were increased in chronic inflammation compared to normal prostate, but no significance was observed when comparing carcinoma vs. normal prostate.

Moreover, we were specifically interested in the association of *HMOX1* and *ANXA2* in the prognosis of PCa. When evaluating the risk of relapse in patients that had undergone radical prostatectomy, results showed that higher levels of expression for both *HMOX1* and *ANXA2* are linked with better RFS and that this correlation remained significant when performing multivariable analyses considering several clinico-pathological parameters. To summarize, high expression of either *HMOX1* or *ANXA2* correlates with longer RFS in PCa patients, and these two genes cooperate in reducing risk of relapse. Considering the well-known features of *ANXA2* as an adhesion molecule and chemoattractant for PCa cells [37], Shiozawa et al. [22] proposed that the lack of *ANXA2* in the primary tumor could exert selective pressure over prostatic cancers favoring not only the detachment of cells from the primary tumor, but also the migration to the bone, a niche rich in ANXA2. Thus, we decided to deepen our analysis focusing on the metastatic progression of PCa. Our results show that databases comprising metastasis information [29] reveal *ANXA2* expression levels to be higher in bone metastatic PCa samples compared with primary PCa tissue. This result is consistent with the increase in *ANXA2* observed in PC3 cells as a consequence of the co-culture with bone cells, indicating that paracrine factors from the bone niche modulate *ANXA2* expression in prostatic tumor cells, probably favoring in this way the colonization and progression of the PCa cells in the skeletal metastatic site. On the other hand, the presence of factors secreted by tumor cells impacted not only on *Anxa2* levels in bone cells, diminishing its gene expression, but also induced a shift of ANXA2 subcellular localization. HO-1 induction in PC3 cells prevented the effect of the co-culture on ANXA2 internalization in Raw264.7 cells, displaying a HO-1 modulatory effect on the interaction between PC3 and bone cells. Interestingly, when analyzing the differential secretomes in the CMs of the co-cultures, uPA was present in the co-culture CM and absent in the co-culture CM when PC3 was pre-treated with hemin. The query against the human and protein databases evidenced that uPA was a human protein and hence was released by the tumor cells. These results together with our previous reports showing a significant downregulation of the uPA/UPAR pathway [16]—resulting in a halted release of uPA—under HO-1 induction in PCa cells, further support the shift in ANXA2 IF staining observed in the osteoclast precursors. When uPA is released to the CM in the co-culture conditions it may trigger the dissemble of the ANXA2⋅P11 tetramer, allowing ANXA2 to internalize and increase its presence in the cytosolic and nuclear compartments. Prostate tumor cells overexpressing HO-1 growing in co-culture with the osteoclastic precursors, appear to reverse this effect. Since no ANXA2 was found in the co-culture secretome, this further suggests the internalization of ANXA2 rather than its release to the CM.

At the endothelial cell surface, ANXA2⋅P11 tetramer acts as a coreceptor for plasminogen and tissue plasminogen activator (tPA), accelerating tPA-dependent activation of the fibrinolytic protease, plasmin, the enzyme that is responsible for thrombus dissolution, and the degradation of fibrin [39]. Plasmin activates pro-MMPs (matrix metallo-proteases) into active MMPs and further activates pro-uPA into active uPA [39]. In addition to the impairment of the uPA/UPAR axis, HO-1 induction in PCa cells has shown to reduce MMP9 expression and activity favoring a less aggressive phenotype [11].

In the presence of uPA, plasmin increases, activating ANXA2 phosphorylation, disassembling the ANXA2⋅P11 tetramer, allowing the ubiquitination and degradation of P11 and the reduced surface level of ANXA2 [40]. This mechanism is described for the macrophage lineage. Of note, osteoclasts are derived from hemopoietic progenitors of the monocyte-macrophage lineage [58], thus is reasonable to infer that the same mechanism occurs in these bone progenitor cells.

## 5. Conclusions

**Figure 7:**
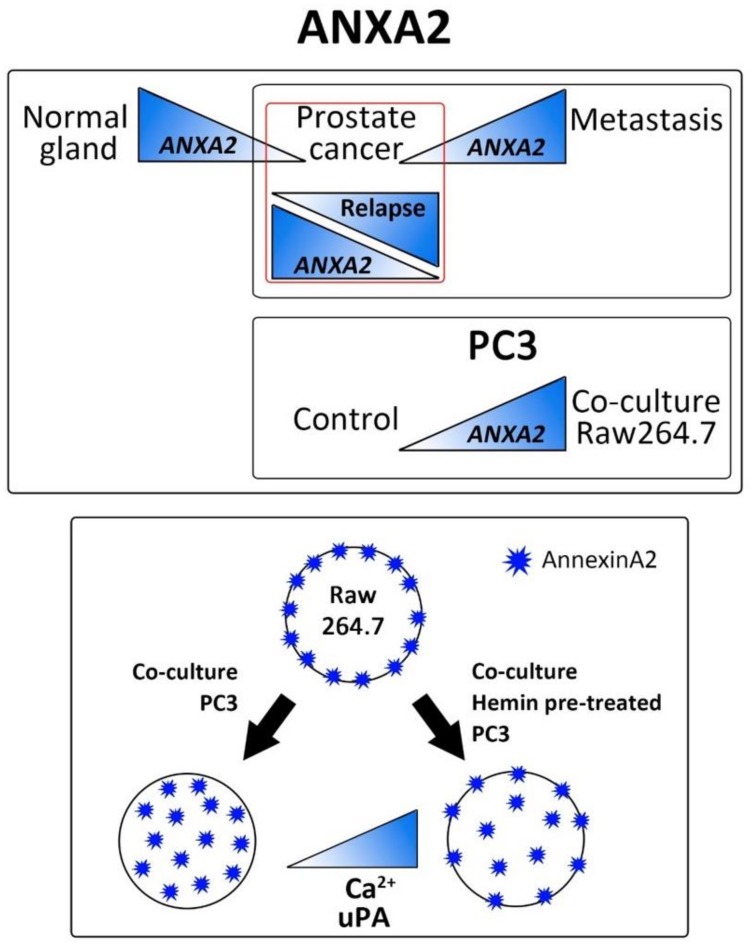
Schematic representation of ANXA2 and HO-1 expression in PCa and metastasis. ANXA2 expression is downregulated in prostate carcinoma compared with the normal gland. In PCa bone metastasis there is an increase in ANXA2 expression compared with the primary tumor site. Similarly, the presence of bone cells in the co-culture induces an increase in ANXA2 expression in the tumor cell, reflecting that the bone niche modulates the expression of ANXA2 in the prostate tumor cell. In turn, the presence of tumor cells triggers a change in the sub-cellular localization of ANXA2 in the pre-osteoclastic cells. This effect is hampered under HO-1 induction in PC3 cells. This phenomenon could be explained, at least in part, by the differences in the availability of free calcium in both conditions and the presence of urokinase (uPA) in the conditioned media.

Altogether, our findings, summarized in Figure 7, point to (1) a shift in *ANXA2* expression when comparing primary prostate tumors with bone metastatic PCa; (2) paracrine factors released from both the tumor and bone niche impacting on ANXA2 expression and signaling, favoring in this way the colonization and progression of the tumor cells in the skeletal metastatic site, and (3) HO-1 modulation in tumor cells, clearly interfering with this signaling.

## Figures and Tables

**Table 1:** Table of employed primers, containing the gene name, specie, sequences, and annealing temperature (Tº an.).

Gene	Species	Primer Fw (5’>3’)	Primer Rv (5’>3’)	T° *an.*
36b4	Mouse	AAGCGCGTCCTGGCATTGTCT	CCGCAGGGGCAGCAGTGGT	60 °C
Anxa2	Mouse	AGGGAGGCTCTCAGCGATAC	TAGGCACTTGGGGGTGTAGA	65 °C
PPIA	Human	GGTATAAAAGGGGCGGGAGG	CTGCAAACAGCTCAAAGGAGAC	60 °C
HMOX1	Human	ACTGCGTTCCTGCTCAACAT	GGGGCAGAATCTTGCACTTT	60 °C
ANXA2	Human	ATATTGCCTTCGCCTACCAG	AGAGAGTCCTCGTCGGTTCCC	65 °C
ANXA2-R	Human	GGCAAAACGGACTCTCTCCT	GAGTCTGTCGGGTTCCTCTG	63 °C

## Supplementary Materials

The following are available online at https://www.mdpi.com/2218-273X/10/3/467/s1, Supplementary Materials and Methods and Figure S1: PC3 cells grown in co-culture with Raw246.7 cells increase invasive properties; Figure S2: Analysis of cell contact density in PC3 cells cultured with conditioned media (CM) from PC3-Raw246.7 co-culture systems; Table S1: List of differential proteins for the conditioned media of PC3 cells co-cultured with RAW264.7 cells identified by LC ESI-MS/MS analysis and matched against human (orange), murine (yellow) or both (green) protein databases; Table S2: List of differential proteins for the conditioned media of hemin pre-treated PC3 cells co-cultured with RAW264.7 cells identified by LC ESI-MS/MS analysis and matched against human (orange), murine (yellow) or both (green) protein databases.
