# Supplementary material for: HO-1 Interactors Involved in the Colonization of the Bone Niche: Role of ANXA2 in Prostate Cancer Progression
Source: Biomolecules. 2020 Mar 18;10(3):467. doi: 10.3390/biom10030467 (PMC7175266; doi:10.3390/biom10030467)
Supplement: Supplementary file 1 [file biomolecules-10-00467-s001.pdf]

## Supplementary Tables and Figures

| Conditioned media from the PC3 and Raw264.7 cells co-culture | | | | | |
|---|---|---|---|---|---|
| Protein | Gene | Theoretical molecular mass (Da) | Coverage % | No. of peptides | PSMs |
| SCO-spondin | SSPO | 547.5 | 0.80 | 1 | 1 |
| Metabotropic glutamate receptor 2 | GRM2 | 95.5 | 4.24 | 1 | 1 |
| Dystonin | DST | 190.2 | 2.33 | 1 | 1 |
| Coiled-coil domain-containing protein 191 | KIAA1407 | 110.5 | 4.38 | 1 | 1 |
| Keratin, type I cytoskeletal 27 | KRT27 | 49.8 | 8.93 | 1 | 1 |
| Baculoviral IAP repeat-containing protein 1 | NAIP | 159.5 | 2.35 | 1 | 1 |
| E3 ubiquitin-protein ligase RNF34 | RNF34 | 4.7 | 77.78 | 1 | 2 |
| Receptor-type tyrosine-protein phosphatase U | PTPRU | 162.3 | 2.14 | 1 | 1 |
| BMP/retinoic acid-inducible neural-specific protein 2 | BRINP2 | 88.9 | 4.34 | 1 | 1 |
| Olfactory receptor 8D2 | OR8D2 | 34.8 | 12.86 | 1 | 1 |
| ATP-dependent RNA helicase DDX18 | DDX18 | 75.4 | 5.82 | 1 | 1 |
| Obscurin | OBSCN | 972.4 | 0.30 | 1 | 1 |
| Neuronal-specific septin-3 | SEPTIN3 | 40.7 | 12.29 | 1 | 1 |
| Urokinase-type plasminogen activator | PLAU | 48.5 | 3.02 | 1 | 1 |
| Ethanolamine-phosphate phospho-lyase | ETNPPL | 55.6 | 8.82 | 1 | 1 |
| Coiled-coil domain-containing protein 65 | CCDC65 | 57.3 | 2.07 | 1 | 1 |
| Immunoglobulin heavy variable 3-53 | IGHV3-53 | 12.8 | 40.52 | 1 | 1 |
| DDB1- and CUL4-associated factor 5 | DCAF5 | 103.9 | 4.56 | 1 | 1 |
| Lamin tail domain-containing protein 2 | LMNTD2 | 70.3 | 5.21 | 1 | 1 |
| Thioredoxin domain-containing protein 11 | TXNDC11 | 110.5 | 3.15 | 1 | 1 |
| Alkylglycerol monooxygenase | AGMO | 51.5 | 9.21 | 1 | 2 |
| Importin subunit alpha-3 | KPNA4 | 57.9 | 5.57 | 1 | 1 |
| Zinc finger protein 585A | ZNF585A | 87.9 | 5.20 | 1 | 1 |
| Ankyrin repeat domain-containing protein 36B | ANKRD36B | 153.5 | 2.37 | 1 | 1 |
| Syntabulin | SYBU | 73 | 5.24 | 1 | 1 |
| Retinoic acid-induced protein 3 | GPRC5A | 40.2 | 10.64 | 1 | 1 |
| CapZ-interacting protein | RCSD1 | 44.1 | 10.44 | 1 | 1 |
| Transmembrane protein 184A | TMEM184A | 46.8 | 9.41 | 1 | 1 |
| Chloride channel protein 2 | CLCN2 | 97.1 | 4.93 | 1 | 1 |
| Enolase-phosphatase E1 | ENOPH1 | 28.6 | 16.34 | 1 | 1 |
| Thymic stromal cotransporter protein | SLC46A2 | 52 | 8.98 | 1 | 1 |
| Protein Gm9923 | - | 67.4 | 6.21 | 1 | 1 |
| DEP domain-containing protein 5 | DEPDC5 | 180.3 | 2.01 | 1 | 1 |
| Protein Fam3c | FAM3C | 27 | 12.15 | 1 | 1 |
| Keratin, type II cytoskeletal 5 | KRT5 | 61.7 | 8.45 | 1 | 1 |
| Ubiquinone biosynthesis protein COQ4 homolog, mitochondrial | COQ4 | 30.1 | 5.26 | 1 | 1 |
| Espin | ESPN | 154.4 | 1.92 | 1 | 1 |
| Dynamin-like 120 kDa protein, mitochondrial | OPA1 | 113.2 | 4.29 | 1 | 1 |
| Striatin-4 | STRN4 | 23.9 | 16.89 | 1 | 1 |
| Transcription factor Dp-2 | DP2 | 42.9 | 11.40 | 1 | 1 |
| Serine/threonine-protein kinase PINK1, mitochondrial | PINK1 | 63.1 | 6.90 | 1 | 1 |
| E3 ubiquitin-protein ligase HERC2 | HERC2 | 527.1 | 0.99 | 1 | 1 |
| Contactin-associated protein 1 | CNTNAP1 | 156.2 | 2.45 | 1 | 1 |
| Cornifin-A | SPRR1A | 15.8 | 25.00 | 1 | 1 |
| GRIP1-associated protein 1 | GRIPAP1 | 95.9 | 3.46 | 1 | 1 |
| Ribonuclease P protein subunit p40 | RPP40 | 41.5 | 9.92 | 1 | 1 |
| Protein Ift140 | IFT140 | 165.8 | 3.07 | 1 | 1 |
| GTP-binding nuclear protein Ran | RAN | 24.3 | 19.91 | 1 | 1 |
| ATPase, H+ transporting, lysosomal V1 subunit B1 | ATP6V1B1 | 56.8 | 6.24 | 1 | 1 |
| Cullin-3 | CUL3 | 88.9 | 5.08 | 1 | 2 |
| Fibrinogen gamma chain | FGG | 52.3 | 1.95 | 1 | 1 |
| Elongation factor 1-alpha 2 | EEF1A2 | 50.4 | 2.38 | 1 | 1 |
| Protein KIAA0100 | KIAA0100 | 253.5 | 1.74 | 1 | 1 |
| Echinoderm microtubule-associated protein-like 4 | EML4 | 110.1 | 3.83 | 1 | 1 |

**Table S1.** List of differential proteins for the conditioned media of PC3 cells co-cultured with RAW264.7 cells identified by LC ESI-MS/MS analysis and matched against human (orange), murine (yellow) or both (green) protein databases.

| Conditioned media from the hemin pre-treated PC3 and Raw264.7 cells co-culture | | | | | |
|---|---|---|---|---|---|
| Protein | Gene | Theoretical molecular mass (kDa) | Coverage % | No. of peptides | PSMs |
| ADP-dependent glucokinase | ADPGK | 54.1 | 9.26 | 1 | 1 |
| Alkylglycerol monooxygenase | AGMO | 51.5 | 9.21 | 1 | 4 |
| ATP-binding cassette sub-family A member 1 | ABCA1 | 13.8 | 31.71 | 1 | 1 |
| AT-rich interactive domain-containing protein 1A | ARID1A | 241.9 | 1.18 | 1 | 1 |
| Bifunctional epoxide hydrolase 2 | EPHX2 | 62.6 | 5.41 | 1 | 1 |
| CD44 antigen | CD44 | 81.5 | 1.62 | 1 | 1 |
| Chloride intracellular channel protein 6 | CLIC6 | 73 | 4.26 | 1 | 1 |
| Collagen alpha-1(XVI) chain | COL16A1 | 157.7 | 2.06 | 1 | 1 |
| Complement component C9 | C9 | 63.1 | 1.61 | 1 | 1 |
| Cytokine-dependent hematopoietic cell linker | CLNK | 49.5 | 4.21 | 1 | 1 |
| E3 ubiquitin-protein ligase HACE1 | HACE1 | 102.3 | 4.40 | 1 | 1 |
| Enoyl-CoA hydratase, mitochondrial | ECHS1 | 31.4 | 12.41 | 1 | 1 |
| Formin-2 | FMN2 | 180 | 1.34 | 1 | 1 |
| Gelsolin | GSN | 85.6 | 1.02 | 1 | 1 |
| Importin subunit alpha-3 | KPNA4 | 57.9 | 5.57 | 1 | 1 |
| Integrator complex subunit 8 | INTS8 | 113 | 3.22 | 1 | 1 |
| Kelch-like protein 26 | KLHL26 | 68.1 | 5.37 | 1 | 1 |
| Keratin, type II cytoskeletal 5 | KRT5 | 23.1 | 19.40 | 1 | 1 |
| Klotho | KL | 116.1 | 2.96 | 1 | 1 |
| Membrane-associated phosphatidylinositol transfer protein 3 | PITPNM3 | 13.1 | 35.34 | 1 | 1 |
| Membrane-spanning 4-domains subfamily A member 7 | MS4A7 | 26.1 | 7.08 | 1 | 1 |
| Methylcrotonoyl-CoA carboxylase subunit alpha, mitochondrial | MCCC1 | 66.4 | 5.02 | 1 | 3 |
| Neuron navigator 2 | NAV2 | 268.1 | 1.12 | 1 | 1 |
| Nuclear receptor-binding protein 2 | NRBP2 | 57.8 | 7.39 | 1 | 1 |
| Nucleoporin SEH1 | SEH1L | 39.6 | 10.56 | 1 | 1 |
| OTU domain-containing protein 1 | OTUD1 | 51 | 6.03 | 1 | 1 |
| Phospholipase B1, membrane-associated | PLB1 | 56.6 | 5.69 | 1 | 1 |
| PRA1 family protein 3 | ARL6IP5 | 21.6 | 14.36 | 1 | 1 |
| Ran-binding protein 10 | RANBP10 | 70.4 | 5.85 | 1 | 1 |
| Retinoic acid-induced protein 3 | GPRC5A | 40.2 | 10.64 | 1 | 1 |
| RNA-binding protein 4 | RBM4 | 40.3 | 10.16 | 1 | 1 |
| Roundabout homolog 3 | ROBO3 | 148.1 | 3.61 | 1 | 1 |
| Scavenger receptor class A member 5 | SCARA5 | 54 | 5.66 | 1 | 2 |
| Thrombospondin-4 | THBS4 | 105.8 | 1.04 | 1 | 1 |
| Tubulin beta-6 chain | TUBB6 | 49.8 | 3.14 | 1 | 1 |
| Tyrosine-protein kinase JAK3 | JAK3 | 125 | 2.22 | 1 | 1 |
| Uncharacterized protein KIAA1551 | KIAA1551 | 194.7 | 2.18 | 1 | 1 |
| Zinc finger and BTB domain-containing protein 17 | ZBTB17 | 87.9 | 4.86 | 1 | 1 |
| Zinc finger protein 544 | ZNF544 | 81.7 | 5.17 | 1 | 1 |
| Retrotransposon gag domain-containing protein 1 | RGAG1 | 144.2 | 1.87 | 1 | 1 |
| Neuronal-specific septin-3 | SEPTIN3 | 40.7 | 12.29 | 1 | 1 |
| Putative deoxyribonuclease TATDN1 | TATDN1 | 33.5 | 7.64 | 1 | 1 |
| Serine/threonine-protein kinase PAK 6 | PAK6 | 74.8 | 6.75 | 1 | 1 |
| Vimentin-type intermediate filament-associated coiled-coil protein | VMAC | 18.3 | 15.38 | 1 | 1 |
| Voltage-dependent L-type calcium channel subunit alpha | CACNA1S | 246 | 0.37 | 1 | 1 |
| Zinc finger CCHC domain-containing protein 5 | ZCCHC5 | 52.8 | 8.84 | 1 | 1 |
| Protein 1600014C10Rik | - | 5.6 | 60.00 | 1 | 1 |
| MCG142225 | - | 34.1 | 8.33 | 1 | 1 |
| MCG134312, isoform CRA_a | - | 77.2 | 5.19 | 1 | 1 |
| MCG142459 | - | 19.5 | 22.94 | 1 | 1 |
| 5'-AMP-activated protein kinase subunit gamma-2 | PRKAG2 | 62.9 | 6.54 | 1 | 1 |
| Acrosin-binding protein | ACRBP | 61.1 | 6.48 | 1 | 1 |
| Adhesion G protein-coupled receptor L4 | ELTD1 | 82.2 | 3.92 | 1 | 1 |
| Angiomotin-like protein 1 | AMOTL1 | 107.9 | 3.62 | 1 | 1 |
| Apolipoprotein A-I | APOA1 | 30.6 | 3.79 | 1 | 1 |
| ATP synthase subunit O, mitochondrial | ATP5O | 23.3 | 5.63 | 1 | 1 |
| Breast cancer anti-estrogen resistance protein 3 | BCAR3 | 92.2 | 4.88 | 1 | 1 |
| Centriolar coiled-coil protein of 110 kDa | CCP110 | 111.1 | 3.39 | 1 | 1 |
| Cytochrome b5 | CYB5A | 15.2 | 32.09 | 1 | 1 |
| F-actin-capping protein subunit alpha-3 | CAPZA3 | 34.9 | 8.03 | 1 | 1 |
| FERM domain-containing protein 3 | FRMD3 | 68.4 | 5.55 | 1 | 1 |
| Furin | FURIN | 86.7 | 3.53 | 1 | 1 |
| Gamma-tubulin complex component 2 | TUBGCP2 | 103.2 | 3.09 | 1 | 1 |
| Netrin-5 | NTN5 | 48.8 | 3.32 | 1 | 1 |
| Protein AF-9 | MLLT3 | 63.3 | 3.51 | 1 | 1 |
| Putative E3 ubiquitin-protein ligase UNKL | UNKL | 11 | 35.19 | 1 | 1 |
| Rho GTPase-activating protein 1 | ARHGAP1 | 54.4 | 9.39 | 1 | 1 |
| SHC-transforming protein 1 | SHC1 | 62.6 | 8.12 | 1 | 1 |
| SID1 transmembrane family member 2 | SIDT2 | 94.4 | 4.93 | 1 | 1 |
| Sodium-dependent neutral amino acid transporter B(0)AT3 | SLC6A18 | 69.2 | 4.88 | 1 | 1 |
| Tubulin beta-4B chain | TUBB4B | 49.8 | 3.15 | 1 | 1 |
| Tubulin polyglutamylase TTLL4 | TTLL4 | 132.4 | 2.77 | 1 | 1 |
| Type 1 phosphatidylinositol 4,5-bisphosphate 4-phosphatase | TMEM55B | 30 | 17.25 | 1 | 1 |
| Unconventional myosin-Ixb | MYO9B | 240.2 | 2.07 | 1 | 1 |
| Vesicle-associated membrane protein 2 | VAMP2 | 17.9 | 28.83 | 1 | 1 |
| YTH domain-containing family protein 3 | YTHDF3 | 63.9 | 8.03 | 1 | 1 |
| Zinc finger protein 536 | ZNF536 | 141.5 | 3.61 | 1 | 1 |
| MANSC domain-containing protein 4 | MANSC4 | 36.8 | 11.87 | 1 | 1 |
| Olfactory receptor 24 | OLFR24 | 34.8 | 8.63 | 1 | 1 |
| Microtubule-associated serine/threonine-protein kinase 3 | MAST3 | 11.1 | 33.02 | 1 | 1 |
| Uncharacterized protein C11orf84 homolog | C11orf84 | 41.2 | 10.24 | 1 | 1 |
| Protein Myo15b | MYO15B | 332.8 | 0.79 | 1 | 1 |
| Type-1B angiotensin II receptor | AGTR1 | 40.9 | 6.96 | 1 | 1 |
| Carbonyl reductase [NADPH] 2 | CBR2 | 25.9 | 14.75 | 1 | 1 |
| Opioid growth factor receptor-like protein 1 | OGFRL1 | 52.2 | 7.33 | 1 | 1 |
| Protein Scml2 | SCML2 | 106.7 | 4.02 | 1 | 1 |
| Protein Zfp108 | ZFP108 | 73.1 | 4.83 | 1 | 1 |
| Core-binding factor, runt domain, alpha subunit 2, translocated to, 3 | CBFA2T3 | 67.9 | 6.61 | 1 | 1 |
| Tripartite motif-containing protein 43B | TRIM43B | 52.2 | 5.17 | 1 | 1 |
| Testis-expressed sequence 11 protein | TEX11 | 109.6 | 2.85 | 1 | 1 |
| Differentially expressed in FDCP 6 homolog | DEF6 | 73.9 | 1.43 | 1 | 1 |
| Vigilin | HDLBP | 141.4 | 2.13 | 1 | 1 |
| A-kinase anchor protein SPHKAP | SPHKAP | 186.3 | 2.41 | 1 | 1 |
| Alpha-enolase | ENO1 | 47.1 | 5.53 | 1 | 1 |
| CREB-binding protein | CREBBP | 265.2 | 3.36 | 2 | 2 |
| Elongation factor 1-alpha 2 | EEF1A2 | 50.4 | 2.38 | 1 | 1 |
| GDNF family receptor alpha-2 | GFRA2 | 51.5 | 7.54 | 1 | 1 |
| Peptidyl-prolyl cis-trans isomerase A | PPIA | 18 | 8.48 | 1 | 1 |
| Phosphoglycerate mutase 1 | PGAM1 | 28.8 | 8.27 | 1 | 1 |
| Probable ATP-dependent RNA helicase DDX59 | DDX59 | 68.8 | 5.49 | 1 | 1 |
| Prosaposin receptor GPR37L1 | GPR37L1 | 52.7 | 7.07 | 1 | 1 |
| Zinc finger protein 608 | ZNF608 | 162.1 | 2.45 | 1 | 1 |
| Triosephosphate isomerase 1 | TPI1 | 30.8 | 3.50 | 1 | 1 |
| 60S ribosomal protein L30 | RPL30 | 12.8 | 24.35 | 1 | 1 |
| Calcium/calmodulin-dependent protein kinase kinase 2 | CAMKK2 | 64.7 | 2.38 | 1 | 1 |

**Table S2.** List of differential proteins for the conditioned media of hemin pre-treated PC3 cells co-cultured with RAW264.7 cells identified by LC ESI-MS/MS analysis and matched against human (orange), murine (yellow) or both (green) protein databases.

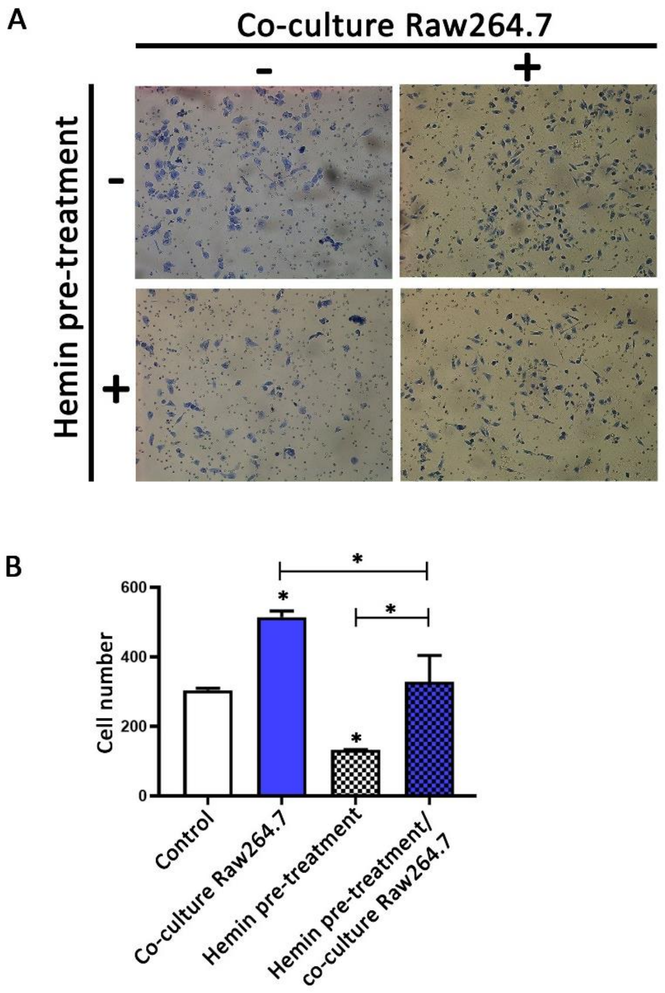


**Figure S1.** PC3 cells grown in co-culture with Raw246.7 cells increase invasive properties. **(A)** PC3 cells pre-treated or not with hemin were plated on Matrigel in a transwell insert and incubated alone or in co-culture with Raw264.7 cells. **(B)** Cell invasion was measured using Matrigel-coated transwell inserts. Cells that had invaded to the underside of the inserts after 24 h of incubation were counted by light microscopy. Four fields of view from each insert were counted. One representative from at least three independent experiments is shown (columns, mean of eight fields of view; bars, SEM; $*p < 0.05$, significant difference).

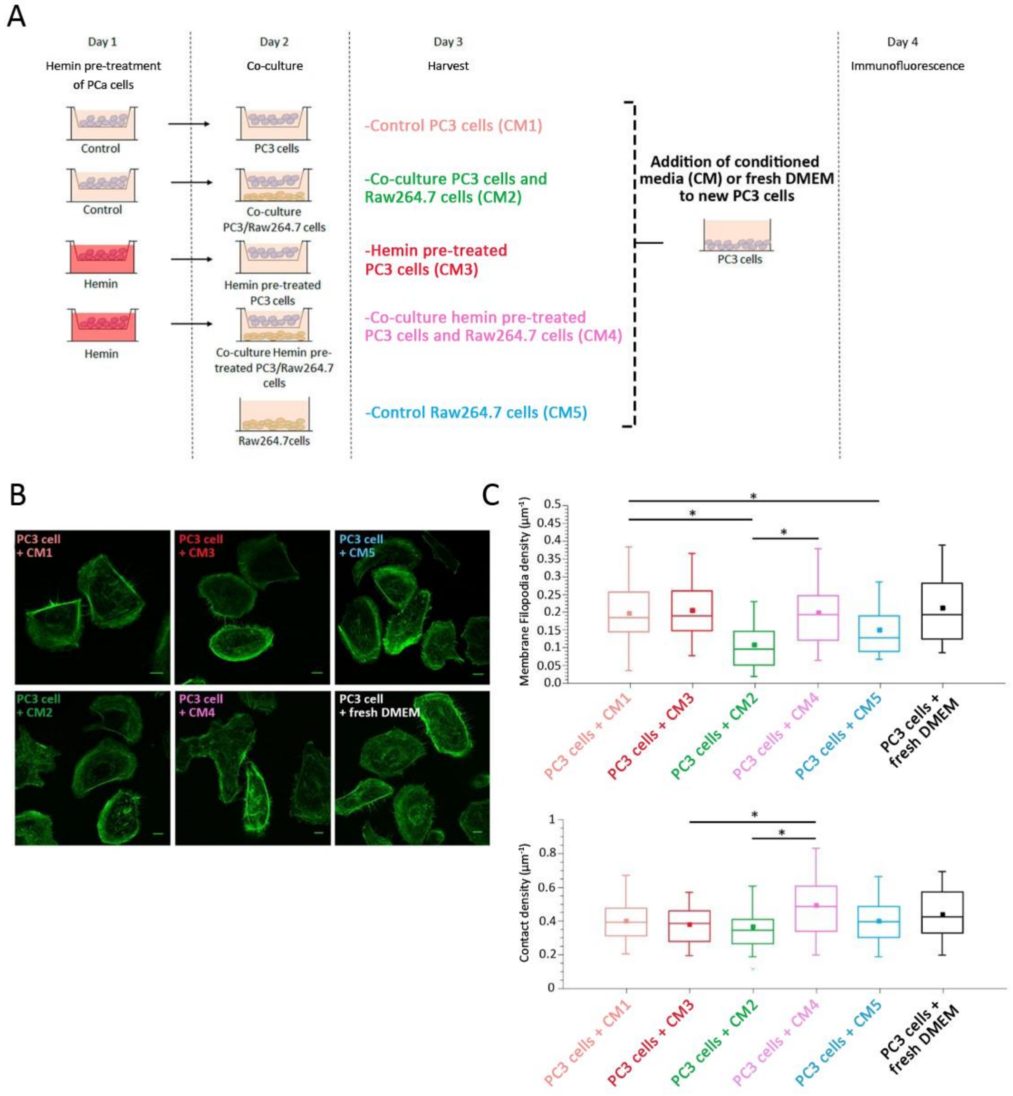


**Figure S2.** Analysis of cell contact density in PC3 cells cultured with conditioned media (CM) from PC3-Raw246.7 co-culture systems. PC3 cells were treated or not with hemin (50 µM, 24 h) and then co-cultured with or without Raw246.7 cells (24 h), in different compartments (insert membrane and well). CM from the different experimental conditions was then added to PC3 cells for 24 h. **(A)** Schematic representation of the experimental design. **(B)** Cells were fixed and stained with rhodamine–phalloidin and imaged by confocal microscopy to assess contact density and number of protrusions (scale bars: 10 µm). One representative image for each group is shown. **(C)** Boxplot comparing the measured filopodia density per cell for the different experimental conditions. Error bars represent standard error ($n \geq 36$ cells per condition; $*p < 0.001$). CM1 (pink): CM from PC3 grown alone; CM2 (green): CM from PC3-Raw246.7 co-culture; CM3 (red): CM from PC3 grown alone pre-treated with hemin; CM4 (purple): CM from PC3 pre-treated with hemin and co-cultured with Raw246.7; CM5 (light blue): CM from Raw246.7 grown alone (upper panel). Boxplot comparing cell–cell contacts densities for the different experimental conditions. An intensity profile for each of these sectors was determined using a custom-made algorithm to count contacts (Matlab) ($n \geq 37$ contact regions per condition; $*p < 0.001$) (lower panel).
